# Skin residual bilirubin volume: a physiologically informed framework for transcutaneous bilirubin interpretation in neonates

**DOI:** 10.3389/fped.2026.1815793

**Published:** 2026-05-14

**Authors:** H. O. Amadi

**Affiliations:** Department of Bioengineering, Imperial College London, London, United Kingdom

**Keywords:** low-resource settings, neonatal jaundice, non-invasive monitoring, phototherapy, recovery value flip (RVP), skin residual bilirubin volume (SRBV), transcutaneous bilirubinometry

## Abstract

**Background:**

Transcutaneous bilirubinometry (TcB) is increasingly relied upon for neonatal jaundice management in settings without access to laboratory-based total serum bilirubin (TSB) testing. However, inconsistent agreement between transcutaneous bilirubin levels (TBL) and TSB limits its reliability as a standalone clinical tool.

**Objective:**

To propose and evaluate skin residual bilirubin volume (SRBV) as a physiological explanation for TcB–TSB discordance and to assess its impact on TcB interpretation during diagnosis, treatment, and recovery.

**Methods:**

This observational, multicentre study was conducted in resource-limited neonatal care settings. TcB readings were calibrated against laboratory TSB in non-jaundiced neonates (TSB <3 mg/dL). Neonates undergoing phototherapy were monitored using paired TcB measurements obtained immediately after treatment interruption (TBL-out) and prior to treatment resumption (TBL-return). TSB was measured before treatment, during treatment, and prior to discharge where feasible. Patterns of TcB–TSB disparity and the “Recovery Value Flip (RVP)” phenomenon were analysed.

**Results:**

TBL consistently equalled or exceeded TSB in cases with paired measurements. Early in treatment at higher bilirubin levels, TBL-return exceeded TBL-out, indicating persistent cutaneous bilirubin. A reproducible RVP was observed, after which TBL-return became lower than TBL-out, coinciding with sustained bilirubin decline. SRBV contribution varied with bilirubin level and treatment phase.

**Conclusion:**

SRBV provides a biologically plausible explanation for TcB–TSB discordance and dynamic TcB behaviour. Incorporating SRBV into TcB interpretation enables physiologically informed monitoring, improving safety and reliability in laboratory-limited neonatal settings.

## 1 Introduction

Neonatal jaundice, the clinical manifestation of elevated bilirubin in newborns, is one of the most common conditions encountered in postnatal care worldwide (1). Physiologic jaundice occurs in the majority of term and preterm infants during the first week of life, affecting up to 60%–85% of neonates and contributing substantially to hospital readmission and healthcare utilisation globally (2, 3). Severe or untreated hyperbilirubinemia can lead to acute bilirubin encephalopathy, long-term neurodevelopmental sequelae, and even death, particularly in low-resource settings where timely diagnosis and treatment are challenging (4, 5).

Accurate measurement of bilirubin levels is essential for identifying neonates at risk and guiding phototherapy decisions. The gold standard for bilirubin quantification remains total serum bilirubin (TSB) obtained via laboratory analysis of blood samples (1). However, in many low- and middle-income countries (LMICs) and remote or resource-limited health facilities, access to laboratory services is often unavailable, delayed, or prohibitively expensive. This limitation reduces timely initiation of therapy and increases the risk of bilirubin toxicity (6).

To address these challenges, transcutaneous bilirubinometry (TcB) was developed as a non-invasive, point-of-care alternative that estimates bilirubin by measuring skin reflectance. TcB devices have been shown to correlate with TSB in many settings and are widely used to screen for neonatal hyperbilirubinemia. Moreover, universal TcB screening has been demonstrated to be more effective than visual inspection alone in identifying neonates requiring intervention, reducing unnecessary invasive sampling, and aiding discharge planning (7).

Despite these advantages, several studies have identified important limitations in the reliability and accuracy of TcB measurements. Early investigations reported that although TcB correlates with TSB, it cannot consistently predict absolute serum bilirubin values, particularly at higher concentrations, and is therefore limited as a standalone diagnostic tool. TcB has generally been recommended as a screening rather than a definitive diagnostic modality, with careful selection of decision thresholds to minimise false-negative results (8). In addition to known variability at higher bilirubin levels and during phototherapy, TcB accuracy may also be influenced by skin pigmentation through optical absorption and scattering effects, further complicating its interpretation as a direct surrogate of serum bilirubin (9–11).

Phototherapy—the mainstay of jaundice treatment—introduces further complexity to TcB interpretation. Some studies have found that TcB measurements during or soon after phototherapy may be less accurate due to changes in skin reflectance and bilirubin distribution, with reports of only moderate correlation with TSB during these phases. Others have shown that even when measured after phototherapy, TcB may underestimate serum levels or demonstrate poor agreement, underscoring the need for laboratory confirmation when available (9–11).

Factors such as skin pigmentation, measurement site, gestational age, and device type have also been shown to influence TcB accuracy and agreement with TSB, limiting universal application without correction or contextual understanding (10).

These persistent inconsistencies in agreement between transcutaneous bilirubin levels (TBL) and TSB have limited TcB's adoption for diagnostic decision-making, treatment monitoring, and discharge planning in settings without laboratory access. This gap highlights the need for approaches that not only improve measurement accuracy but also contextualise TcB readings within the underlying bilirubin physiology—especially in environments where laboratory tests are scarce.

This study addresses a fundamental question: why does transcutaneous bilirubinometry behave inconsistently, and how can it be made more reliable without additional equipment?

## 2 Conceptual framework: TSB, TBL, and SRBV

### 2.1 Defining skin residual bilirubin volume (SRBV)

**Figure 1:**
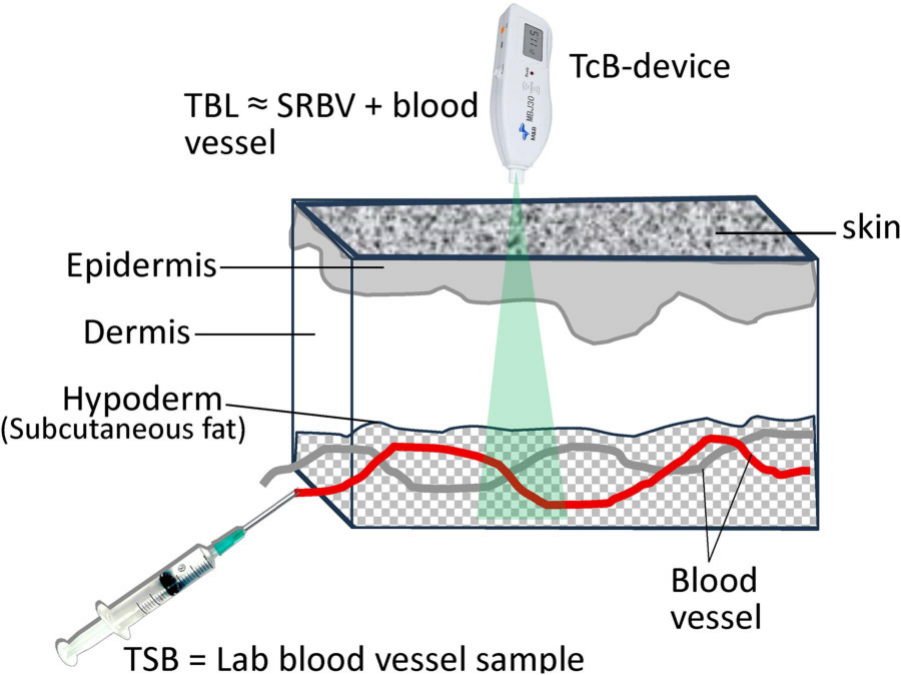
TSB, TBL, SRBV concept.

TSB represents the absolute concentration of bilirubin within the bloodstream, measured invasively via blood sampling. In contrast, TBL is obtained using optical techniques that interrogate bilirubin within and beyond the intravascular compartment (Figure 1).

We propose that TcB devices inherently measure:

- Intravascular bilirubin (corresponding to TSB), plus
- Residual bilirubin retained in the skin and subcutaneous tissues, termed Skin Residual Bilirubin Volume (SRBV)

Thus:

TBL≈TSB+SRBV

In neonates with very low bilirubin concentrations (TSB <3 mg/dL), bilirubin remains intravascular and SRBV ≈ 0. Under these conditions, TBL closely approximates TSB.

At higher bilirubin levels, bilirubin diffuses into the skin, generating a non-zero SRBV and causing TBL to exceed TSB.

### 2.2 Hypothetical pathophysiology

Bilirubin extravasation into the skin produces visible jaundice and reflects a dynamic equilibrium between serum and tissue compartments. The threshold at which this occurs varies between patients, influenced by skin thickness, perfusion, maturity, and prior bilirubin exposure.

Consequently, identical TSB values may correspond to different derived SRBV magnitudes in different neonates, particularly during recovery following phototherapy. This variability provides a physiological basis for TcB–TSB discordance.

## 3 Materials and methods

### 3.1 Study design and setting

This was a prospective, multicentre observational study conducted across 10 grassroots mission hospital facilities in the southeastern region of Nigeria as part of an ongoing task-shift implementation programme for neonatal jaundice management in laboratory-limited settings (12). The study focused on evaluating the physiological basis and clinical interpretability of transcutaneous bilirubin (TcB) measurements during neonatal jaundice treatment.

### 3.2 Transcutaneous bilirubinometer calibration

The MBj20 transcutaneous bilirubinometer (Beijing M&B Electronic Instruments, Beijing, China) was calibrated locally at each participating facility prior to clinical deployment. Calibration was performed using paired transcutaneous bilirubin level (TBL) and laboratory-based total serum bilirubin (TSB) measurements obtained from neonates without clinical jaundice (TSB <3 mg/dL).

For calibration, TcB measurements were obtained from the forehead concurrently with venous blood sampling for laboratory TSB analysis. Paired values were used to derive a centre-specific calibration coefficient, which was applied to the TcB device before routine clinical use at that facility. This approach was intended to minimise device and site-related measurement variability and to ensure consistency across centres.

### 3.3 Study objectives

The study was designed to address the following objectives:

1. To establish whether TBL measurements are consistently equal to or greater than TSB across patients and bilirubin severity categories.
2. To characterise the magnitude of skin residual bilirubin volume (SRBV) across clinically relevant bilirubin ranges.
3. To examine SRBV behaviour near treatment completion, particularly when TBL values fall below 11 mg/dL.
4. To determine whether SRBV exhibits time-dependent behaviour during phototherapy and recovery.

### 3.4 Treatment protocol

Neonates diagnosed with jaundice received phototherapy using the solar-powered PoliteUltraLumen phototherapy device, which was deployed uniformly across all participating mission hospitals (13). Treatment irradiance was standardised across centres according to the PoliteUltraLumen deployment protocol to minimise treatment-related variability. Phototherapy was delivered in 3-hour continuous treatment sessions, separated by 20-minute breaks to allow feeding and rehydration. Patients wore blindfold eyepatch that extends to the forehead during treatment sessions.

### 3.5 Monitoring and data collection

During each treatment break, TcB measurements were obtained using the calibrated MBj20 device at a standardised anatomical site (forehead) and recorded as:

- TBL-out: measured immediately at cessation of a phototherapy session
- TBL-return: measured immediately before resumption of the subsequent session

Laboratory blood samples for TSB quantification were collected before initiation of treatment, at least once during mid-treatment, and prior to discharge, in accordance with the clinical protocol at each centre. These paired TcB and TSB measurements were used to assess TcB–TSB disparity and infer SRBV dynamics during treatment and recovery.

### 3.6 Ethical considerations

Ethical approval for the study was obtained from the Ethics Committee of the Catholic Diocese of Nsukka, Nigeria. Written informed consent was obtained from the parent or caregiver of each neonate prior to enrolment. All procedures were conducted in accordance with applicable ethical standards and local clinical governance requirements.

## 4 Results

### 4.1 Study population and consistency of TBL ≥ TSB

A total of 102 neonates were recruited across six of the ten participating grassroots mission hospitals (Table 1), covering a wide spectrum of jaundice severity with baseline transcutaneous bilirubin levels ranging from 6.8 to 30.4 mg/dL. Due to financial constraints, three centres were unable to perform laboratory TSB measurements, and among these, only one facility completed full TSB sampling as specified in the protocol, while the remaining centres obtained partial TSB data.

**Table 1:** Site-level characteristics, RVP manifestation, and availability of paired TSB measurements.

Hospital	TBL-TSB behavioural values							
	Total patient	Mean post-natal Age (±sd, days)	Mean pre-treatment TBL (mg/dL)	TBL range (mg/dL)	Mean TBL decline rate (mg/dL/h)	Paired TSB & TBL availability^a^	End-treatment TBL ≥ TSB (%)^b^	RVP Observed in cases^c^
BSH Nsukka	23	5.7 (2.4)	14.03	10–28	0.27	Complete	100%	91%
SPH Abakaliki	23	3.8 (1.6)	12.37	8.9–18.4	0.52	Limited	-	100%
MJH Okigwe	10	5.7 (3.3)	12.29	10.4–14.9	0.23	Limited	-	100%
IHH Urualla	20	2.7 (2.1)	8.97	6.8–14.3	0.21	Limited	-	71%
MCH Enugu	13	3.8 (1.8)	16.33	10.7–23.5	0.18	Partial	-	92%
IHMH Nkpor	13	3.7 (2.2)	15.22	8.3–30.4	0.18	Complete	100%	100%

BSH, Bishop Shanahan Hospital; MCH, Mother of Christ Hospital; IHMH, Immaculate Heart of Mary Hospital; SPH, St Patrick Hospital; MJH, Mbano Joint Hospital; IHH, Immaculate Heart Hospital. RVP (Recovery Value Flip) was defined using paired transcutaneous measurements (TBL-out vs. TBL-return) and assessed independently of laboratory TSB availability.

a Paired TSB availability indicates the extent of laboratory serum bilirubin sampling alongside TcB measurements at each centre: Complete (all patients), Partial (subset of patients), Limited (minimal sampling). Limitations were primarily due to financial constraints in participating centres.

b End-treatment TBL ≥ TSB (%) represents the proportion of paired measurements in which transcutaneous bilirubin equalled or exceeded serum bilirubin. This analysis was restricted to cases with available paired TSB data.

c RVP reproducibility was consistent in high TBL cases (>10 mg/dL).

Across all bilirubin categories in which paired measurements were available, transcutaneous bilirubin levels (TBL) were consistently equal to or greater than corresponding total serum bilirubin (TSB) values. This relationship was observed irrespective of centre, baseline bilirubin level, or treatment stage. In several patients, the magnitude of TBL–TSB disparity increased discretely with rising bilirubin levels. Early TBL measurements below 3 mg/dL demonstrated negligible TBL–TSB difference, whereas higher baseline values were associated with measurable disparities, reflecting inter-individual variability.

Table 1 summarises recruitment by centre, baseline bilirubin ranges, average pre-treatment TBL values, observed TBL decline rates during treatment, and the frequency with which end-treatment TBL values remained equal to or greater than TSB.

### 4.2 Recovery value flip (RVP) during phototherapy

Serial TBL measurements obtained during phototherapy revealed a reproducible dynamic pattern among neonates with baseline TBL values greater than 9 mg/dL. Early in treatment, TBL measured immediately at the end of a phototherapy session (TBL-out) was consistently lower than TBL measured immediately before the subsequent session (TBL-return). This pattern was observed across centres and treatment sessions.

After several phototherapy cycles, a Recovery Value Flip (RVP) was observed, characterised by a reversal of this relationship such that TBL-return became consistently lower than TBL-out (Table 1). Following the occurrence of RVP, TBL declined steadily until treatment completion. The RVP phenomenon was clearly identifiable in 92% of patients with adequate serial TcB data and was observed across a range of baseline bilirubin severities.

**Figure 2:**
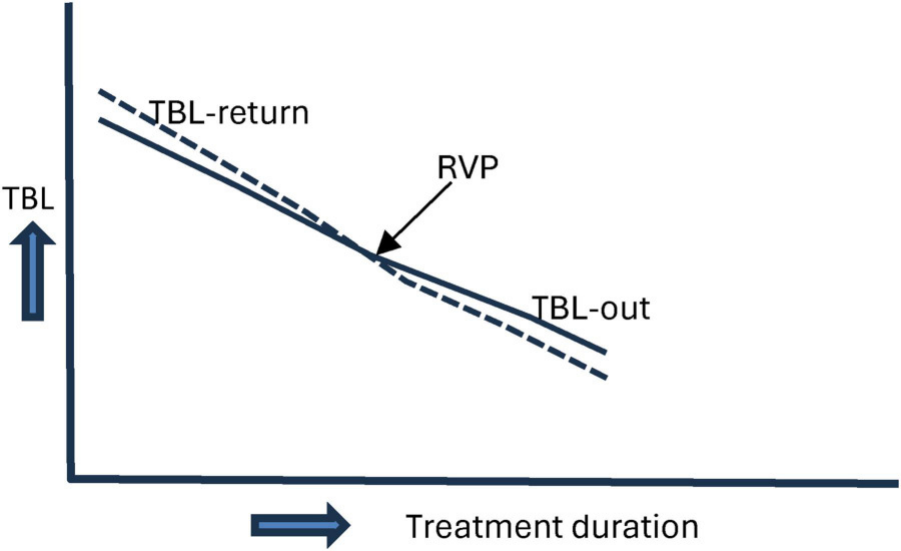
Representative transcutaneous bilirubin trajectories illustrating the recovery value flip (RVP) phenomenon and subsequent decline during phototherapy.

Figure 2 illustrates representative TBL trajectories demonstrating the RVP phenomenon and the subsequent monotonic decline in bilirubin levels until discharge.

### 4.3 Fractional contribution of SRBV to measured TBL

The estimated fractional contribution of skin residual bilirubin volume (SRBV) to measured TBL is summarised in Table 2, stratified by bilirubin severity category and treatment stage. SRBV was expressed as a proportion of TBL and averaged within clinically relevant bilirubin ranges.

**Table 2:** Estimated average fractional contribution of skin residual bilirubin volume (SRBV) to measured transcutaneous bilirubin (TBL) before treatment and during recovery.

Treatment stage	%SRBV in TBL					
	Pre-treatment			End-treatment		
Hospital/GROUP	<9 mg/dL (±sd)	9–11.5 mg/dL (±sd)	11.6–15 mg/dL (±sd)	<9 mg/dL (±sd)	9–11.5 mg/dL (±sd)	11.6–15 mg/dL (±sd)
BSH Nsukka (*n* = 46)	NA	30% (0.02)	9% (0.04)	17% (0.09)	27% (0.07)	24% (0.03)
MCH Enugu (*n* = 8)	NA	11% (0.02)	12% (0.05)	NA	NA	NA
IHMH Nkpor (*n* = 12)	NA	NA	NA	21% (0.05)	11% (0.04)	28% (0.03)
Average		20%	11%	19%	19%	26%

BSH, Bishop Shanahan Hospital; MCH, Mother of Christ Hospital; IHMH, Immaculate Heart of Mary Hospital; NA, no data captured, *n*, total number of paired blood samples in dataset.

At pre-treatment, measurable SRBV contribution was observed primarily in neonates with mild to moderate jaundice. The average SRBV fraction was 20% (SD 0.02) in the 9–11.5 mg/dL group and 11% (SD 0.05) in the 11.6–15 mg/dL group. In neonates with TBL <9 mg/dL, SRBV was not detectable, and insufficient data were available to reliably estimate pre-treatment SRBV in neonates with TBL >15 mg/dL.

During the recovery stage, SRBV contribution was detectable across a broader range of bilirubin values. Neonates with TBL <9 mg/dL demonstrated a mean SRBV fraction of 19% (SD 0.07). In the 9–11.5 and 11.6–15 mg/dL groups, mean SRBV fractions increased to 19% (SD 0.05) and 26% (SD 0.03), respectively. These fractional estimates were observed consistently across centres where sufficient paired data were available. RVP was observed in all evaluated cases across centres based on TcB dynamics, whereas TcB–TSB agreement analyses were limited to sites with available paired laboratory measurements (Table 1).

## 5 Discussion

### 5.1 Interpreting transcutaneous bilirubinometry through SRBV

This study reframes transcutaneous bilirubinometry from a flawed surrogate of serum bilirubin into a composite physiological signal. The persistent observation that transcutaneous bilirubin levels (TBL) were equal to or greater than total serum bilirubin (TSB) across bilirubin strata supports the conceptual model in which TcB reflects the sum of intravascular bilirubin and skin residual bilirubin volume (SRBV). Apparent TcB “overestimation,” particularly at higher bilirubin levels and during phototherapy, therefore, reflects physiological bilirubin persistence within the skin rather than device inaccuracy.

Importantly, this relationship was dynamic rather than static, evolving across treatment phases. The observed variability in TBL–TSB disparity between patients is consistent with inter-individual differences in bilirubin extravasation, cutaneous deposition, and clearance, reinforcing the need for interpretation frameworks that extend beyond isolated numeric thresholds.

### 5.2 Clinical meaning of the recovery value flip (RVP)

Serial TcB monitoring during phototherapy revealed a reproducible phenomenon termed the Recovery Value Flip (RVP). Early in treatment, TBL measured before therapy resumption (TBL-return) exceeded values measured immediately after phototherapy cessation (TBL-out), consistent with ongoing bilirubin redistribution into the skin during treatment breaks. As treatment progressed, a distinct inflection point was reached at which TBL-return became lower than TBL-out, signalling depletion of the cutaneous bilirubin reservoir and dominance of sustained bilirubin clearance.

The consistency of the RVP across centres and patients suggests that it represents a clinically meaningful physiological milestone rather than random measurement variability. The RVP therefore provides a non-invasive marker of treatment adequacy that is particularly valuable in settings where laboratory confirmation is unavailable. However, at low pre-treatment bilirubin values, TcB measurement more closely approximates serum bilirubin and dynamic features such as the RVP may be attenuated, reflecting reduced cutaneous bilirubin contribution rather than inconsistency of the additive model.

### 5.3 SRBV as a unifying explanation for TcB–TSB discordance

By explicitly accounting for SRBV, this study provides a physiological explanation for the long-recognised discordance between TcB and TSB reported across devices, populations, and treatment contexts. Rather than indicating poor device performance, TcB variability appears to encode information about bilirubin compartmentalisation and redistribution.

The graded SRBV fractions observed across bilirubin strata and treatment stages (Table 2) further support this interpretation. SRBV derivative emerged with increasing bilirubin burden, persisted during recovery, and declined only after sustained treatment response, confirming TcB as a biologically interpretable measurement rather than a direct serum analogue. The dataset from this study presents the relationship TcB ≈ TSB + SRBV as a context-dependent physiological model, rather than an absolute rule. Situations in which TcB < TSB—as has been reported previously in the literature (9–11)—may reflect measurement-site differences, optical artefacts, or rapid intravascular bilirubin shifts not yet reflected in the cutaneous compartment. Therefore, this study emphasises that SRBV—as a derivative—is an interpretive framework grounded in observed patterns, and that deviations reported in the literature qualify for further investigation within this model.

The SRBV construct is consistent with established principles of bilirubin distribution, including reversible binding to tissue components and delayed equilibration between vascular and extravascular compartments. Bilirubin movement from the bloodstream into the skin is likely governed by diffusion gradients, protein binding, and local tissue characteristics, resulting in time-dependent accumulation and clearance. These processes provide a plausible mechanistic basis for the observed lag between serum bilirubin decline and TcB normalisation.

### 5.4 SRBV-adjusted TcB interpretation as a proposed standard of care

**Figure 3:**
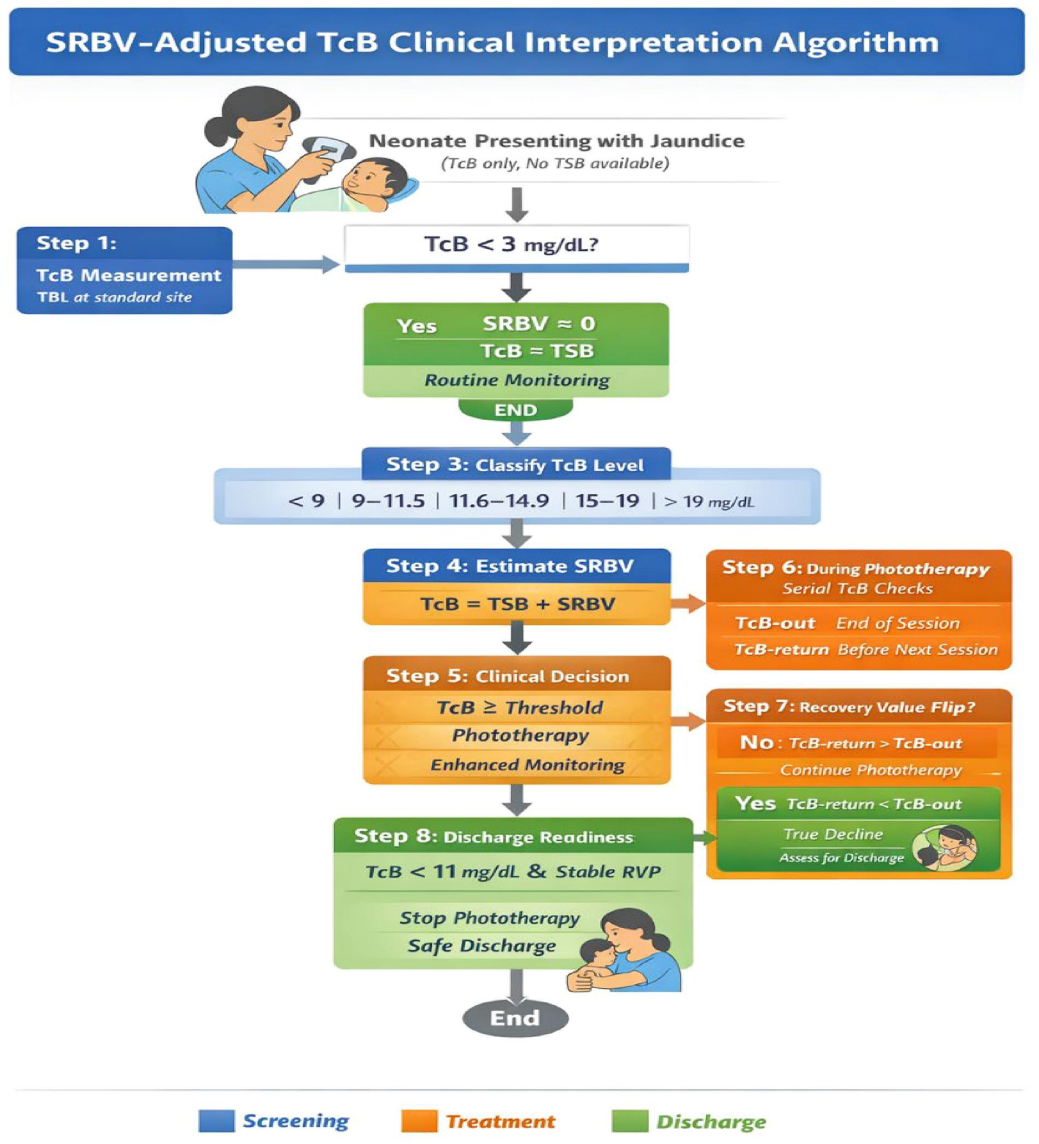
SRBV-adjusted transcutaneous bilirubin interpretation algorithm.

Based on these findings, I propose an SRBV-adjusted TcB interpretation framework for use in laboratory-limited neonatal care settings (Figure 3). This approach departs from reliance on isolated TcB values and instead emphasises contextual, trend-based interpretation incorporating treatment phase and SRBV dynamics.

Under this proposed standard of care:

1. TcB values <3 mg/dL reliably approximate serum bilirubin, reflecting negligible SRBV.
2. TcB values ≥3 mg/dL are assumed to include a variable SRBV component and should not be interpreted as direct serum equivalents.
3. Paired TcB measurements during phototherapy breaks inform compartment dominance:
  - TBL-return > TBL-out indicates SRBV persistence.
  - Observation of the RVP (TBL-return < TBL-out) signals depletion of cutaneous bilirubin.
4. Post-RVP TcB trends are prioritised over absolute values for treatment continuation, cessation, and discharge decisions.

Within this framework, the RVP functions as a non-invasive physiological marker of recovery, enabling safer TcB-guided care without laboratory confirmation. While SRBV is inferred from systematic TcB–TSB divergence and dynamic TcB behaviour rather than directly measured, the reproducibility, directionality, and treatment-phase dependence of these patterns support its interpretation as a physiologically meaningful construct rather than a measurement artefact.

The non-linear trajectory of TcB decline during phototherapy—characterised by more rapid early reduction followed by attenuation—has been previously well-described and this is consistent with biphasic bilirubin kinetics as found in this study. Within this derivative SRBV framework, this pattern may reflect initial clearance of circulating bilirubin followed by slower mobilisation of bilirubin retained within cutaneous compartments. The Recovery Value Flip (RVP) observed in this study may represent a clinically detectable transition between these phases.

### 5.5 Implications for neonatal care in laboratory-limited settings

As outlined in the Introduction, neonatal jaundice continues to cause preventable morbidity and mortality in settings where access to laboratory-based TSB testing is limited or delayed. The proposed SRBV-adjusted TcB interpretation framework directly addresses this gap by extending the clinical reliability of TcB in contexts where its use is unavoidable.

#### 5.5.1 Implications for clinical practice

By accounting for skin-related bilirubin persistence, this approach

- Enables structured interpretation of TcB as a composite physiological signal rather than a direct surrogate of serum bilirubin
- Supports safer TcB-guided decision-making in settings without access to laboratory TSB measurement
- Facilitates task-shifting by providing a standardised, physiology-informed framework for nurses and community healthcare providers
- Improves interpretation of treatment response during phototherapy, including identification of the Recovery Value Flip (RVP)
- Reduces unnecessary blood sampling and potential overtreatment through trend-based TcB assessment
- Strengthens the clinical utility of solar-powered and decentralised phototherapy application, and for efficient jaundice treatment in resource-limited environments
- Shortens hospital stays

### 5.6 Positioning SRBV within existing guidelines

Current neonatal jaundice guidelines appropriately caution against standalone TcB use at higher bilirubin levels and during phototherapy. The SRBV framework provides a physiological rationale for these cautions while offering a pathway to overcome them in constrained settings. By explicitly accounting for cutaneous bilirubin persistence and redistribution, SRBV-adjusted TcB interpretation may serve as an interim or complementary standard of care where laboratory confirmation is unavailable. The concept of bilirubin distribution beyond the intravascular compartment has been previously recognised, including early work by Rubaltelli et al., which highlighted tissue-associated bilirubin and its clinical implications (14, 15). The SRBV framework extends this understanding by providing a clinically observable and operational interpretation of this compartment through serial transcutaneous measurements, thereby linking established physiology with bedside monitoring.

Skin pigmentation is a recognised modifier of TcB accuracy through its effects on optical absorption and scattering (9–11). While the relatively homogeneous study population may have reduced intra-cohort variability, melanin-related effects cannot be excluded and may contribute to inter-individual differences. However, the use of paired and serial within-patient measurements in this study mitigates the influence of static pigmentation factors, supporting the interpretation of observed TcB dynamics as primarily physiological. Postnatal age represents another additional physiological determinant of bilirubin dynamics, with levels typically rising to a peak before declining. Variation in age at presentation may therefore influence both baseline bilirubin burden and the relative contribution of derived SRBV, as well as the timing and expression of other TcB-derived features such as the RVP. These factors should be considered when interpreting TcB trends in clinical practice.

### 5.7 Limitations and future directions

This study has several limitations. Findings were derived from a limited number of TcB devices, and device-specific optical characteristics may influence SRBV behaviour. Measurements were obtained predominantly from the forehead, and regional variation in skin properties may affect SRBV expression at alternative sites. SRBV was quantified indirectly from systematic TcB–TSB disparity rather than measured as an absolute tissue parameter, constraining mechanistic precision. In addition, partial laboratory data availability limited assessment of population-level modifiers such as gestational age. Skin pigmentation as a potential confounder was not assessed, hence future validation is needed across diverse populations, skin tones, and device types to further disentangle optical from physiological contributions to TcB measurements.

This study did not observe enough sample population for a Bland–Altman analysis, which would have been valuable for assessing agreement between two measurement techniques. However, the primary aim of this early-stage observational study was not to establish interchangeability between TBL and TSB, but rather to propose and explore a physiological model explaining their systematic divergence. Given the incomplete availability of paired TSB measurements across centres, and the conceptual focus on SRBV as a dynamic component of TcB measurement, a formal Bland–Altman analysis was not feasible within the current dataset. A larger sample population is hence an important aspect for future work.

This study involved the application of one model of phototherapy system across centres within southeastern Nigeria only. Further multicentre validation across diverse populations, devices, and clinical environments is required. Formal correlation between irradiance and RVP timing was beyond the scope of this study and hence not investigated. This should be explored in future controlled investigations with deliberately varied irradiance levels or other devices. Nevertheless, the consistency of observed TcB–TSB relationships and treatment-phase dynamics establishes SRBV as a clinically meaningful construct and provides a rational foundation for the proposed interpretation framework.

## 6 Conclusion

This study introduces skin residual bilirubin volume (SRBV) as a unifying physiological construct explaining the systematic disparity between transcutaneous and serum bilirubin measurements, particularly at higher bilirubin levels and during phototherapy. By recognising TcB as a composite of intravascular and skin-deposited bilirubin, the SRBV framework reframes TcB variability from a limitation into clinically interpretable information. Integration of SRBV into a structured interpretation algorithm extends the role of TcB beyond screening to treatment monitoring and discharge decision-making in laboratory-limited settings. By operationalising tissue-associated bilirubin as a measurable component of TcB, the SRBV derivative framework extends earlier physiological observations into a clinically actionable model for neonatal jaundice management. While further validation is required, SRBV represents a pragmatic and scalable advance aligned with the realities of neonatal care delivery in resource-constrained environments.

### 6.1 Significance statement

Transcutaneous bilirubinometry is widely used in low-resource settings but has been limited by poor agreement with serum bilirubin at clinically relevant levels. This study introduces skin residual bilirubin volume (SRBV) as a physiological explanation for this disparity and proposes an SRBV-adjusted interpretation framework that transforms TcB variability into actionable clinical information. The approach advances safer, non-invasive jaundice management in settings where reliance on TcB is a necessity rather than a choice.

## Data Availability

The raw data supporting the conclusions of this article will be made available by the authors, without undue reservation.
